# PhyloCloud: an online platform for making sense of phylogenomic data

**DOI:** 10.1093/nar/gkac324

**Published:** 2022-05-11

**Authors:** Ziqi Deng, Jorge Botas, Carlos P Cantalapiedra, Ana Hernández-Plaza, Jordi Burguet-Castell, Jaime Huerta-Cepas

**Affiliations:** Centro de Biotecnología y Genómica de Plantas, Universidad Politécnica de Madrid (UPM) and Instituto Nacional de Investigación y Tecnología Agraria y Alimentaria (INIA-CSIC), 28223 Madrid, Spain; Centro de Biotecnología y Genómica de Plantas, Universidad Politécnica de Madrid (UPM) and Instituto Nacional de Investigación y Tecnología Agraria y Alimentaria (INIA-CSIC), 28223 Madrid, Spain; Centro de Biotecnología y Genómica de Plantas, Universidad Politécnica de Madrid (UPM) and Instituto Nacional de Investigación y Tecnología Agraria y Alimentaria (INIA-CSIC), 28223 Madrid, Spain; Centro de Biotecnología y Genómica de Plantas, Universidad Politécnica de Madrid (UPM) and Instituto Nacional de Investigación y Tecnología Agraria y Alimentaria (INIA-CSIC), 28223 Madrid, Spain; Centro de Biotecnología y Genómica de Plantas, Universidad Politécnica de Madrid (UPM) and Instituto Nacional de Investigación y Tecnología Agraria y Alimentaria (INIA-CSIC), 28223 Madrid, Spain; Centro de Biotecnología y Genómica de Plantas, Universidad Politécnica de Madrid (UPM) and Instituto Nacional de Investigación y Tecnología Agraria y Alimentaria (INIA-CSIC), 28223 Madrid, Spain

## Abstract

Phylogenomics data have grown exponentially over the last decades. It is currently common for genome-wide projects to generate hundreds or even thousands of phylogenetic trees and multiple sequence alignments, which may also be very large in size. However, the analysis and interpretation of such data still depends on custom bioinformatic and visualisation workflows that are largely unattainable for non-expert users. Here, we present PhyloCloud, an online platform aimed at hosting, indexing and exploring large phylogenetic tree collections, providing also seamless access to common analyses and operations, such as node annotation, searching, topology editing, automatic tree rooting, orthology detection and more. In addition, PhyloCloud provides quick access to tools that allow users to build their own phylogenies using fast predefined workflows, graphically compare tree topologies, or query taxonomic databases such as NBCI or GTDB. Finally, PhyloCloud offers a novel tree visualisation system based on ETE Toolkit v4.0, which can be used to explore very large trees and enhance them with custom annotations and multiple sequence alignments. The platform allows for sharing tree collections and specific tree views via private links, or make them fully public, serving also as a repository of phylogenomic data. PhyloCloud is available at https://phylocloud.cgmlab.org

## Graphical Abstract

**Graphical abstract:**
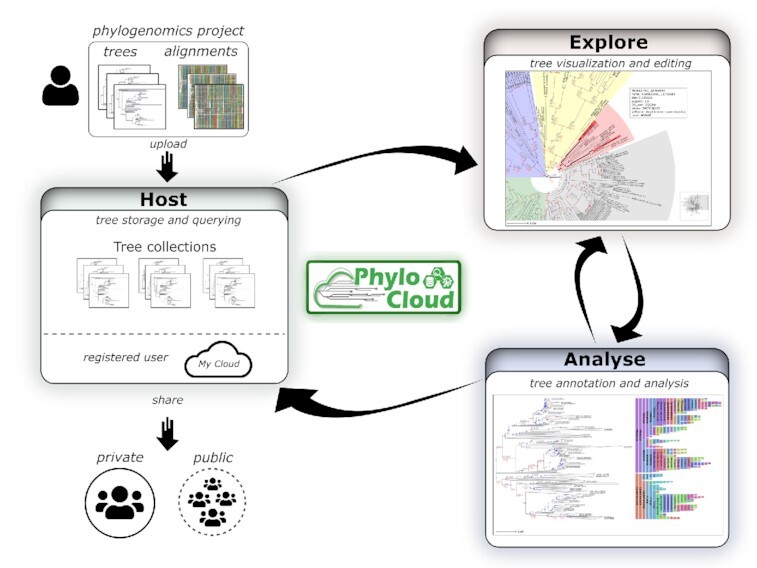
PhyloCloud is an online platform aimed at hosting, organizing, sharing, exploring and analyzing trees and alignments from their phylogenomics projects.

## INTRODUCTION

Phylogenomics is a relatively recent field aiming at applying evolutionary analyses at the genomic scale (1). Most common applications include establishing phylogenetic relationships between species based on multiple genes (2), predicting gene function (3), studying the evolutionary history of protein families (4), detecting horizontal gene transfer events (5) and tracing the origin of neo-functionalization events (6). To this end, phylogenomic studies not only rely on the computation of phylogenetic trees, but also on their analysis and interpretation. Extracting meaningful information out of raw phylogenies, usually encoded in Newick or NEXUS formats, involves specialized analyses and operations such as automatic tree rooting, detection of duplication events, calculation of distances between tree nodes or topology comparisons (7). Many of these tasks can only be performed through *ad hoc* bioinformatic solutions, including command line tools (e.g. Newick Utilities (8)) and specialized programming libraries (9–12). In addition, to make sense out of phylogenetic trees, it is usually required that the different tree nodes are annotated with functional, taxonomic or any other type of relevant metadata, which can be used to enrich graphical representations of the results. In fact, graphical exploration of annotated trees is a crucial step in the phylogenetic workflow, often required to identify and interpret evolutionary patterns.

Advanced visualization tools exist on different platforms that assist in the creation of custom layouts and graphical representations. Most notably, iTOL (13) provides numerous graphical options for online interactive visualization of large annotated trees; ETE-Toolkit (12) provides programmatic annotation and visualization of trees using the Python programming language, and ggtree (14) offers custom visualizations for the R programming environment. Additionally, many stand-alone and online tree visualization programs are available that allow for the interactive exploration of trees using different styles and representations (e.g. among the most popular, FigTree, Dendroscope (15), PhyD3 (16), Phylo.io (17)). However, such tools depend on previously annotated trees and tend to require substantial work to adjust custom tree visualization layouts. Most importantly, current phylogenomic studies can produce hundreds of trees along with their corresponding multiple sequence alignments (MSAs), which can also be large, hitting the limits of many of these programs.

Here, we present PhyloCloud (https://phylocloud.cgmlab.org), an online platform aiming at providing an integrative framework for interactive analysis, annotation, visualization and management of phylogenetic trees, regardless of their number or size. PhyloCloud allows users to upload, organise and share both public and private collections of phylogenetic trees, annotate them using predefined methods, and explore them using built-in visualization layouts. Additionally, the platform provides several handy tools for comparative genomics, such as querying information from the NCBI Taxonomy and GTDB databases (18,19), comparing tree topologies in a graphical manner, and quickly building phylogenetic trees using custom data. Notably, PhyloCloud’s tree visualization capabilities are built upon the latest developments of the ETE software (12), which enables fast interactive exploration of huge trees, while providing advanced graphical features and adaptive zooming.

## RESULTS

### Storing, managing and sharing tree collections

PhyloCloud organizes tree uploads by collections, following a similar approach as the iTOL online viewer (13). Although single trees can also be submitted, the tree upload form allows users to submit up to 1000 trees in Newick or Extended Newick format in batch mode, together with their MSAs in FASTA format. Once loaded, all trees are assigned to a new or existing collection, and their content (i.e. node names) is automatically indexed to enable quick text-based searches. Moreover, trees under a given collection can be easily browsed, expanded and rearranged between collections. By default, users can create collections anonymously, and these can be shared with colleagues through a private link. Optionally, users can register into PhyloCloud for better organizing their collections and fine controlling the sharing options. There is currently no limit on the number of trees that can be assigned to a collection, enabling PhyloCloud as a custom repository of data analogous to project-oriented databases (20,21) As an example, PhyloCloud currently features several public tree collections covering up to 112,563 gene trees from recently published phylogenomic studies (22,23)

### Tree annotation and analysis

**Figure 1.**
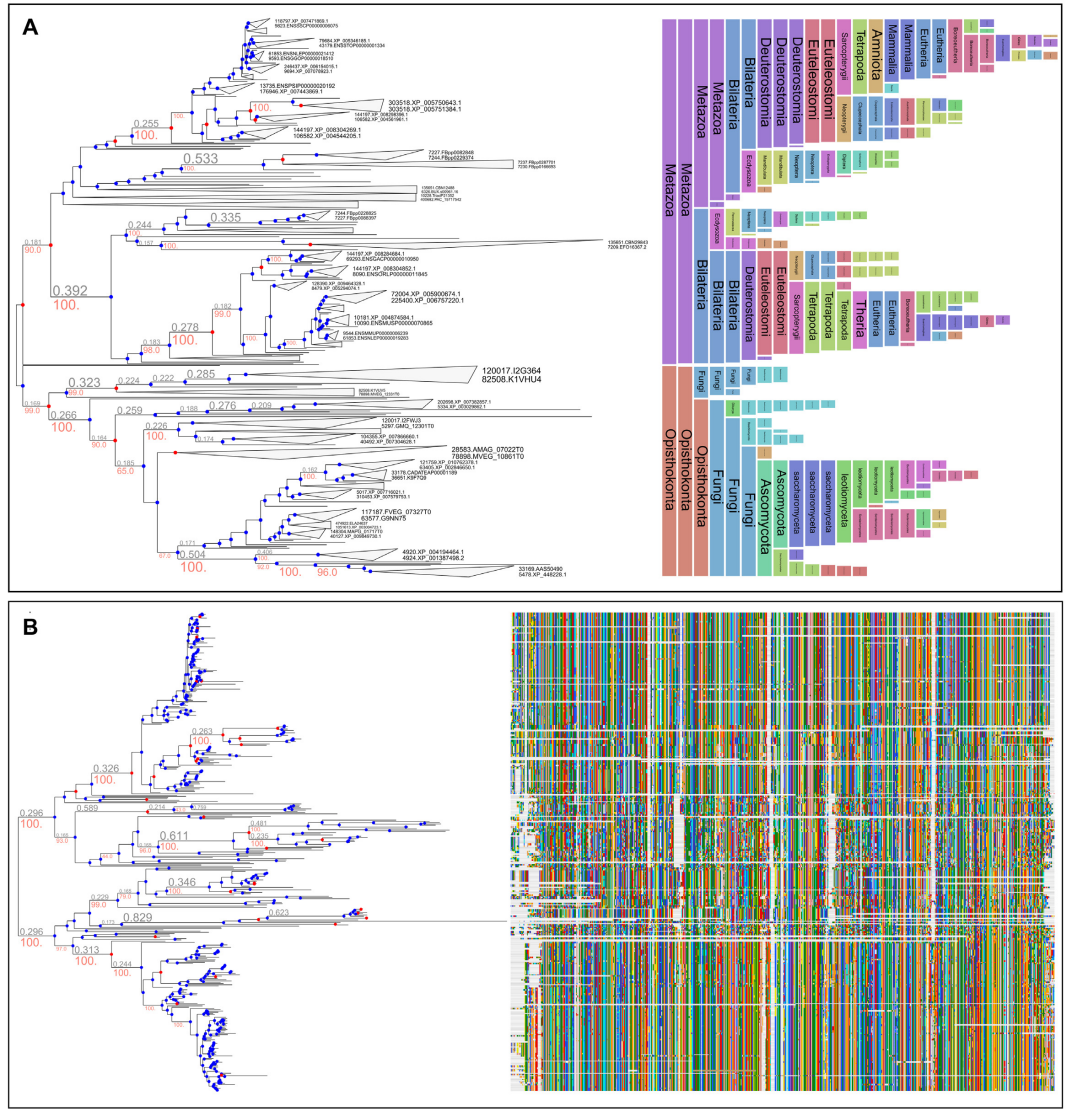
Alignment and taxonomic annotation panels. (**A**) A phylogenetic tree shown along with NCBI taxonomic labels inferred for each internal branch, corresponding to the last common ancestor rank and lineage of its descendants. Speciation (dots in blue) and duplication (dots in red) events were automatically detected and annotated on internal nodes. (**B**) A phylogenetic tree is visualized along with the condensed representation of the MSA used to reconstruct the phylogeny. All three panels (tree, alignments and taxonomic annotations) can be shown together and are synchronized and dynamically adjusted to the zoom level and browsing region of the tree image.

Various tree annotation and analysis options are provided in PhyloCloud. First, any species or gene tree with NCBI or GTDB v202 species identifiers in their leaf names can be automatically annotated with taxonomic information. This includes not only the assignment of scientific names and full lineage paths to leaf nodes but also the annotation of internal branches with the last common ancestor lineage of their descendants. Such annotations are automatically recognized and can be displayed in the tree explorer panel as vertical color bands at the right side of the tree image, thus guiding the exploration of large trees (Figure 1A). Moreover, based on the species content of each internal node, PhyloCloud can identify duplication and speciation events in gene trees using the species overlap detection algorithm (24) (Figure 1, blue and red nodes), inferring also pairwise orthology relationships between tip nodes. These options are enabled solely by extracting the species identifiers associated with each leaf in the tree, which can be parsed automatically from their names, or manually adjusted using the graphical node editing options.

### Link to multiple sequence alignments

One of PhyloCloud’s primary features is the availability of linking MSAs to their corresponding trees. By doing so, the interactive MSA panel can be activated alongside with the phylogenetic tree visualization (Figure 1B). By doing so, the dynamic exploration of tree topologies is synchronized with a graphical and also dynamic representation of the sequences associated to each leaf node. When tree nodes are collapsed, one or more representative sequences from their descendants are displayed, enabling smooth interactive visualisation of large datasets.

### Tree visualisation, editing and searching

**Figure 2.**
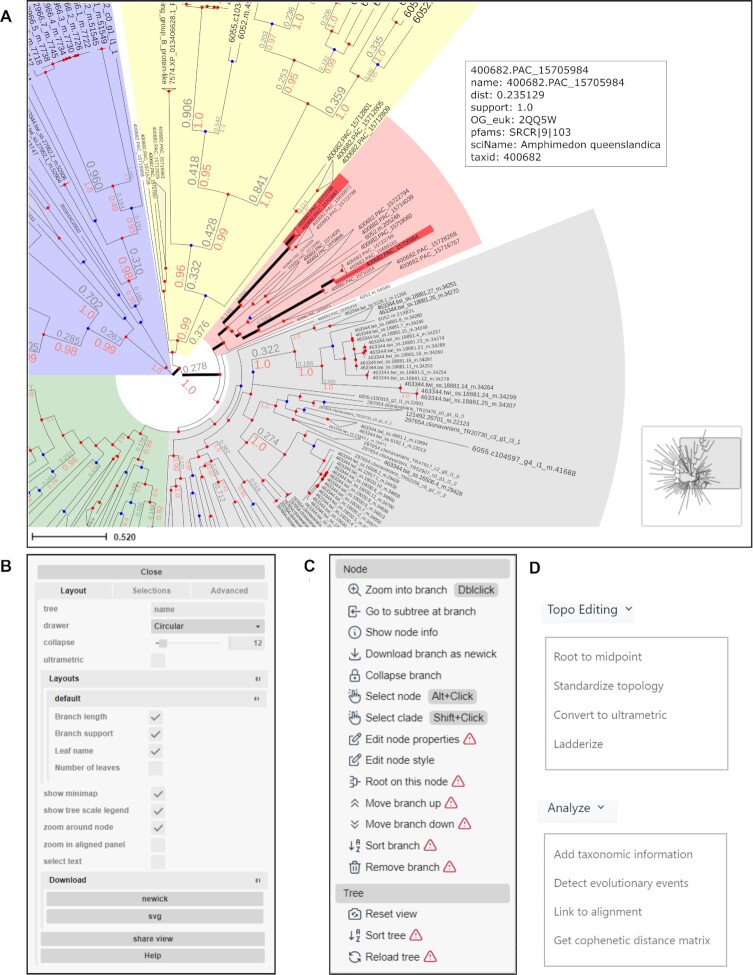
Overview of the PhyloCloud tree explorer interface. (**A**) A phylogenetic tree from the Spongilla featured collection, visualized in circular layout. Some clades were shaded with different background colors. Support values (red) and branch lengths (grey) are displayed on top of branches and collapsed nodes are shown as a triangle summarizing the length of underlying branches. Hits from two searches are highlighted with thicker black lineage branches and bright red tips. The annotations of one of the hits are shown on the top right corner. The minimap (bottom right) facilitates navigation. (**B**) The control panel allows users to customise visualization layout and features, and to perform text-based searches. (**C**) The node editor panel provides access to node-specific actions, such as creating subtrees, collapsing, pruning, rooting and more. (**D**) Drop-down menus showing the topological and analytic actions which can be performed on the current tree in PhyloCloud.

PhyloCloud uses the new visualization framework implemented in ETE 4.0, which allows for the interactive exploration of huge phylogenies based on a context-based adaptive zooming strategy (Figure 2). Moreover, the interactive tree explorer allows users to perform various editing options on specific nodes or the tree as a whole, including topology modifications such as automatic re-rooting, pruning, ladderizing branches, resolving polytomies or converting tree topology into an ultrametric (Figure 2B, C and D). These changes can be either discarded after testing or saved permanently into the database. In addition, specific subtrees can be extracted from particular clades and exported separately in Newick format, including node annotations. Notably, the tree explorer allows for quick searches within large tree topologies, where each search can be associated w a different label and color (Figure 2, red branches in panel A). This provides a quick view on the distribution of specific features.

### Quick phylogenetic reconstruction

Although phylogenetic reconstruction is not the main focus of PhyloCloud, the platform provides access to a quick click-and-go phylogenetic reconstruction workflow based on those available from the command line tools included in ETE v3.2.1. To ensure relatively fast results, the available workflows offer two aligners (MAFFT (25) and Clustal Ω (26)), several MSA trimming options (trimAl 2.0 (27)), and FastTree for approximate likelihood tree inference (28). Input data, which can be either nucleotide or amino acids sequences, are expected in FASTA format. Results include MSA files (also in FASTA format) and phylogenetic tree files (Newick format), which can be quickly inspected in place, or saved into a PhyloCloud collection for further sharing and analysis. Phylogenetic results obtained from more advanced workflows like the ones available in other platforms such as NGPhylogeny (29) can also be loaded into PhyloCloud.

### Comparing tree topologies

PhyloCloud provides online access to the tree comparison capabilities implemented in the ETE Toolkit library, enabling the possibility of highlighting the topological differences between two trees of medium size. Besides providing general metrics such Robinson-Foulds distance and the percentage of identical branches found in both trees, PhyloCloud allows users to explore, side-by-side, which branches in one tree are different from the other. To this end, the visualization panel is synchronized between both trees allowing users to quickly identify which is the closest match of a given branch, as well as the euclidean distance to it. The algorithm used for comparing tree topologies is based on minimizing the overall Euclidean distance between all branches in both trees.

### Querying taxonomic databases

Querying taxonomic databases is a common task in phylogenetics and other evolutionary analyses. PhyloCloud provides a convenient interface to retrieve full lineage information and subtrees from the NCBI and GTDB taxonomy databases. Thus, users can query with either NCBI or GTDB taxonomic identifiers and obtain a pruned and fully annotated tree including all the descendants of the queried clade.

## CONCLUSIONS

PhyloCloud is an online platform that combines analytic tools, utilities and advanced visualization options relevant for a wide range of phylogenetic and phylogenomic studies. PhyloCloud focuses on simplicity and aims at allowing the use of commonly used workflows by non-expert users. The platform is, by design, intended for massive datasets, providing novel solutions for the exploration of large phylogenetic trees and multiple sequence alignments, serving also a global repository for hosting and sharing public phylogenomics datasets. We expect future improvements to provide new analytic workflows, annotation layouts, and sharing capabilities.
